# Occlusion of the Os Uteri

**Published:** 1851-11

**Authors:** E. G. Mygatt

**Affiliations:** Richmond, McHenry Co.


ARTICLE III.

*Occlusion of the Os Uteri.* By E. G. Mygatt, M. D.

On the 31st of August last I was called to see Mrs. —— aged 40, of the sanguine temperament, and a good constitution, in labor with her first child. She had lived with her first husband twelve years and with the present one eight, and this as she informed me was her first pregnancy. The pains were short with long intervals. An examination showed the Uterus to be high up and the Os Uteri not reached.

In the course of twenty-four hours the pains had become strong and frequent, recurring every three or four minutes; patient vomiting occasionally; free from headache or frebile excitement; pulse quiet; skin of the natural temperature; said she felt the motions of the child. Examinations now showed a round, soft, smooth, imperforate tumor or sac. It was soft and fluctuating in the absence of pain, very easily carried before the finger as far as it could be introduced, but dense though elastic during pain and brought within half a finger's length of the vulva.

On the morning on the next day, Sept. 2d, my friend Dr. H——, who has been above thirty years in practice was requested to see my patient. He however, found himself as much in the fog as I had been, as no trace or resemblance of an Os Uteri could be found.

As there was no rigidity of the presenting part and only a slight acceleration of the pulse, we did not bleed our patient but agreed to empty the bowels, keep her quiet, and see what time would develope. Thus passed another twenty-four hours, when we called in consultation Dr. S——, an experienced practitioner who made an examination without knowing our opinion. He informed us that he was unable to find an Os Uteri.

As the strength of the patient remained good, and the fœtus not impacted, it was agreed to quiet the pains with Morphine and mitigate her sufferings with Chloroform and wait further time.

The pains were now irregular, occurring with force and then ceasing altogether for hours. I had previously instituted a thorough examination with the index and middle fingers introduced, changing hands that I might ascertain with certainty that the vagina was attached to the presenting part all around. Perhaps it may not be quite orthodox to use or recommend the introduction of the middle finger along with the index, but where the Os Uteri is high up or far back we may gain at least half an inch in this way without any inconvenience to the patient. It enabled me in this case to ascertain that no part of the child engaged fairly in the superior strait.

I had noticed in my examinations a slight sulcus or groove about half an inch long on the lower portion of the presenting part. It felt something like a depressed cicatrix, apparently solid and impervious, whilst the surface immediately around it was as smooth and soft as any other portion.

On the morning of the fifth day the use of the Vaginal Speculnm was proposed, that we might bring it to bear upon the little furrow, and ascertain its nature ; this was objected to on the part of the patient.

During the afternoon of the same day the pains were strong and propulsive, and while we were consulting as to the necessity and propriety of an operation, we were notified that something was passing. It appeared to be a fluid without much fetor, resembling bloody pus. Some 6 or 8 ounces passed during half an hour. I now sat down to my patient and pressed rather firmly on the sulcus during a pain with the point of my finger, when to my surprise and delight the adhesion gave way and permitted my finger to pass the hitherto occluded Os Uteri ; the pains were now effective ; dilatation rapid; the breech presented and a still born child of the average size was delivered about two hours after the opening of the Os Uteri.

Nothing unfavorable has occurred since her delivery, but she has had a good "getting up," better than the average of cases.

All the evidence that could be gathered respecting inflammation of the Os and Cervix Uteri is that she suffered rather more than is usual from Dysuria during the early part of her pregnancy and the husband informed me that sexual intercourse had been quite painful to her.

If we had made an early and free incision through the presenting part of the Uterus we should have been justified by good authority and there is some probability that we should have saved the life of the child. It is however, quite uncertain whether it would have been as well for the mother unless the incision had traversed the exact line of the closure.

As this is a rare case, so rare indeed that Dewees, Burns, Denman, and others of great experience and observation, had never witnessed it, I judged it worthy of record in some Medical Journal.

*Richmond, McHenry Co., Sept. 22d*, 1851.

